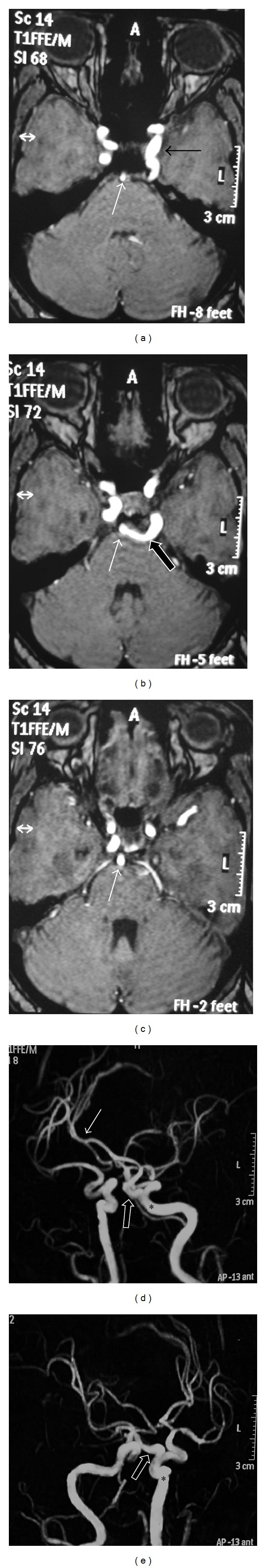# Persistent Primitive Trigeminal Artery: An Unusual Cause of Vascular Tinnitus

**DOI:** 10.1155/2013/275820

**Published:** 2013-12-29

**Authors:** Ananya Panda, Arundeep Arora, Manisha Jana

**Affiliations:** Department of Radiodiagnosis, All India Institute of Medical Sciences (AIIMS), Ansari Nagar, New Delhi 110029, India

## Abstract

Pulsatile tinnitus is generally of vascular origin and can be due to arterial, venous, or systemic causes. While certain congenital anatomical variants and arterial vascular loops have been commonly found in symptomatic patients undergoing imaging, persistent primitive trigeminal artery in association with isolated tinnitus is unusual. Thus we report a patient with unilateral isolated pulsatile tinnitus who was evaluated with magnetic resonance angiography and was found to have a persistent primitive trigeminal artery. We also briefly discuss vascular tinnitus as well as the embryology, imaging, and classification of persistent primitive trigeminal artery with the clinical implications.

## 1. Introduction

Tinnitus is typically a ringing sound in one or both ears in the absence of an external stimulus. It can either be pulsatile (synchronous with the patient's pulse) or nonpulsatile. It can also be subjective (audible only to the patient) or objective (audible to both the patient and the examiner) [1]. Pulsatile tinnitus is generally of vascular origin and includes arterial, venous, and systemic causes [2]. Among the arterial causes, anatomical variants like aberrant internal carotid artery, laterally displaced carotid arteries, persistent stapedial artery, and vascular loops have a well-known association with tinnitus [3, 4]. We report a patient with an unusual cause of vascular pulsatile tinnitus, namely, persistent primitive trigeminal artery (PPTA), followed by a brief review of vascular tinnitus as well as PPTA.

## 2. Case Report

Our case is a 48-year-old lady who was referred to our department with a long-standing history of unilateral left-sided pulsatile tinnitus for one year. There were no associated complaints such as trigeminal neuralgia, facial weakness, vertigo, or hearing deficits. Clinical and otoscopic examinations were normal. An arteriovenous vascular malformation was suspected, so she underwent a 3-tesla magnetic resonance (MR) imaging of brain and cerebellopontine angle along with a 3D time of flight (TOF) angiography in our hospital. 

MR and MRA base images show a serpiginous vessel (black arrows in Figures 1(a) and 1(b)) arising from the cavernous part of the left internal carotid artery (ICA), coursing posterior, and laterally around the dorsum sellae up to the basilar artery (BA) (white arrows in Figures 1(a) and 1(b)) in the prepontine cistern. This vessel is the primitive persistent trigeminal artery (PPTA) forming an anastomosis between the carotid and the basilar systems. The proximal basilar artery is hypoplastic (white arrow in Figures 1(a) and 1(b)) but increases in calibre beyond the site of anastomosis (white arrow in Figure 1(c)) and divides into its terminal posterior cerebral artery branches. On TOF angiographic projection images (Figures 1(d) and 1(e)), the PPTA forms a “Tau” sign (block arrow) with the ICA (∗) as it courses from ICA to basilar artery (white arrow).

## 3. Discussion

Pulsatile tinnitus is a sound in the ear synchronous with the patient's pulse and can either be subjective or objective. This symptom raises a concern of a vascular tumour like paraganglioma, vascular malformation, or vascular anomaly [1]. Other causes include atherosclerosis, fibromuscular dysplasia, arterial aneurysms and dissections, dural arteriovenous malformations, jugular bulb variants, dural venous thrombosis, idiopathic intracranial hypertension, and systemic diseases leading to hyperdynamic circulation like anemia and thyrotoxicosis [1–3, 5]. Congenital and anatomical variants are found in a substantial subset of patients undergoing imaging for pulsatile tinnitus. Among the vascular variants, vascular loops in the cerebellopontine angle are the most common cause and are proposed to cause neurovascular contact with the eighth nerve leading to tinnitus [6] with microvascular decompression surgeries leading to symptomatic relief [7]. However other studies have shown that there is no significant relationship between these loops and tinnitus [8, 9]. Other congenital variants include aberrant internal carotid artery [8] and persistent stapedial artery which also courses through the middle ear en-route to the middle cranial fossa [10]. Combinations of these two anomalies and bilateral anomalies have also been reported in patients with audiovestibular symptoms [11–13]. 

Both persistent stapedial artery and primitive persistent trigeminal artery represent persistent carotid vertebrobasilar anastomosis, others being persistent hypoglossal and proatlantal arteries. In the developing embryo, there are four paired anastomoses between the internal carotid and the primitive vertebrobasilar systems, namely, the trigeminal, otic, hypoglossal, and the proatlantal intersegmental arteries. These carotid-vertebrobasilar arterial anastomoses begin to regress and obliterate from days 29–35. The first to do so is the otic artery, sequentially followed by the hypoglossal, trigeminal and the proatlantal intersegmental artery. Failure of regression of one or more of these primitive anastomoses leads to persistent carotid-vertebrobasilar anastomoses, of which persistent primitive trigeminal artery is the most common type [14].

The overall incidence of persistent primitive trigeminal artery (PPTA) is 0.1 to 1% [15, 16] which represents 85% of these persistent carotid vertebrobasilar anastomosis. The persistent trigeminal artery usually arises from the cavernous portion of the internal carotid artery and reaches the basilar artery. It can either have a medial, intrasellar, or a lateral, parasellar course. In medial course, it enters the sella, runs in its own, groove and then pierces the dura to join the basilar artery between the origins of anterior inferior cerebellar and the superior cerebellar arteries. In lateral course, it runs along the lateral side of sella, near the sensory root of trigeminal ganglion, and then joins the basilar artery between the origins of anterior inferior cerebellar and superior cerebellar arteries. The persistent trigeminal artery may also terminate at the level of and anastomose directly with either superior cerebellar, anterior, inferior, or posterior inferior cerebellar arteries, which are termed as persistent trigeminal artery variants [16].

The origin, course, and its relationships with the neighbouring structures can be well-delineated by noninvasive MR angiography (MRA) using 3D TOF sequence [17]. The “*Tau” *sign is a frequently cited sign on MR imaging and refers to the appearance of a presellar internal carotid artery (ICA) when a persistent trigeminal artery arises from it on a parasagittal MR image. The configuration of the presellar segment of ICA with the PPTA arising from it represents the Greek letter *τ* (Tau) [18]. In most cases, the basilar artery proximal to the anastomoses is variably hypoplastic and the hypoplastic basilar artery is considered to be an ancillary diagnostic sign.

Angiographically, Saltzman classified PPTA into 3 types depending on the presence or absence of PCom and the status of PCA [19]. More recently, a new MRA based classification has been proposed where, in addition to the original Saltzman descriptions, 2 more types have been included [20]. PPTA can also be associated with aneurysms of circle of Willis, arteriovenous malformations, and fistulas. Recognition of the type of PPTA and associated vascular abnormalities are needed for the optimal diagnostic workup of patients with vertebrobasilar insufficiency. Other therapeutic implications include knowledge prior to endovascular interventions to prevent any risk of embolization from the carotid to posterior circulation during procedures and prior to surgery around sella and cavernous sinus region to prevent inadvertent damage to these arteries leading to haemorrhage and ischemia [20].

While PPTA is often incidentally detected, symptomatic patients can present with trigeminal neuralgia. This is more common with the lateral course of artery which causes neurovascular compression of Gasserian ganglion [15, 21]. Isolated pulsatile tinnitus has only been reported in one case series till date where the trigeminal artery terminated as the posterior inferior cerebellar artery (PICA), thus representing a PPTA variant [22]. Another case reported a patient with an indirect carotid-cavernous fistula in left eye, presenting with pulsatile tinnitus, proptosis, and diplopia, who also had an associated right-sided PPTA variant terminating as PICA. However in this patient, the right PPTA variant was an incidental finding as tinnitus was secondary to the left carotid-cavernous fistula [23]. 

Thus this case differs from the other described cases as our patient had isolated unilateral pulsatile tinnitus and was found to have a same-sided PPTA with its normal anastomosis with basilar artery and without a PPTA variant. There was no other cause to explain pulsatile tinnitus in this patient. While it is uncertain whether the PPTA was an incidental finding in this patient or was the cause of symptom, this conundrum is common in many studies of pulsatile tinnitus [1, 4]. The probable hypothesis to explain tinnitus in this case can be that increased flow through the PPTA produces sound waves, which, due to the proximity of the anomalous vessel to the cerebellopontine angle, are mechanically conducted via the cerebrospinal fluid to the perineural spaces around VIIIth nerve and also to the cochlea via bone conduction causing perception of tinnitus [6]. The reason why tinnitus has not been reported with other cases of PPTA could be either because of the overall rarity of PPTA, due to underreporting or due to the pericarotid venous plexus acting as effective dampeners in those patients [24].

## Figures and Tables

**Figure 1:**